# Mutism as a Symptom of Gender Dysphoria: A Case Report

**DOI:** 10.7759/cureus.67451

**Published:** 2024-08-21

**Authors:** Gemma Espejo, Jacob Albritton, Mahta Baghoolizadeh

**Affiliations:** 1 Psychiatry and Human Behavior, University of California Irvine School of Medicine, Irvine, USA

**Keywords:** voice, mutism, transgender and gender diverse, transgender medicine, transgender care, selective mutism, gender dysphoria (gd)

## Abstract

Transgender individuals may perceive voice as a meaningful component of their gender identity. We present a 33-year-old transgender woman with a history of major depressive disorder and social anxiety disorder who avoided communication through speech due to discomfort with the sound of her voice. While the patient had a history of social anxiety disorder, her symptoms related to speech production appeared rooted in her gender identity. Selective mutism and social anxiety disorder may be present in transgender individuals and affect communication; however, there may be a subset of patients whose mutism is driven by gender dysphoria. Clinicians should be aware that gender dysphoria may influence mutism, as gender-affirming interventions may improve both the psychological and vocal aspects of communication in transgender patients.

## Introduction

Voice plays a significant role in the expression of gender, and transgender individuals may have varying perspectives on the role of their voice in their gender identity and expression. Generally, pitch is the most recognizable difference in gendered voices [1]. Conceptually, voice may be perceived as part of the secondary sexual characteristics described in gender dysphoria in the Diagnostic and Statistical Manual of Mental Disorders, 5th edition, text revision (DSM-5-TR) [2]. While research in this area is limited, transgender patients may use speech therapy to assist with raising vocal pitch or testosterone hormone therapy to assist with lowering pitch [1]. Meanwhile, selective mutism is defined as a consistent failure to speak in social situations, which interferes with educational or occupational achievement or social communication. To meet the criteria for selective mutism, symptoms cannot be better explained by another communication disorder, and they must be present for at least one month [2]. Social anxiety disorder, which may be associated with selective mutism, is characterized by a marked fear of social situations in which one could be judged [2]. We present a case of a transgender patient with mutism and propose that mutism may be a manifestation of gender dysphoria.

## Case presentation

A 33-year-old transgender woman with a history of gender dysphoria, major depressive disorder, and social anxiety disorder presented to the emergency department with worsening suicidal thoughts. The patient endorsed multiple depressive symptoms, including poor sleep, guilt, and low energy. During serial mental status examinations, the patient preferred to communicate through writing. There was no concern for disorganized thought processes, behavior, or catatonic symptoms during admission. The chart indicated a history of mutism, and collateral information from an outpatient provider described long-standing social anxiety characterized by not wanting to show her face or eat in public, in addition to mutism. During admission, it was clarified that the patient had been selectively mute since she "came out" in her twenties when she disclosed to others that she identifies as female.

Regarding gender-affirming care, hormone replacement therapy was initiated five months prior to admission, and a bilateral orchiectomy was performed two months later. The patient was also self-administering estradiol valerate 10 mg intramuscularly weekly. She expressed a desire to pursue voice modification therapy in the future. Notably, the patient reportedly had difficulty maintaining her medical care, particularly with a psychotherapist, due to mutism, as many points of care were frequently conducted over the phone.

Throughout admission, the patient predominantly communicated through writing and spoke minimally, with only four instances of verbal communication during her four-day stay. During the encounter with the most verbal output, she described feeling disgusted by the sound of her voice. On the unit, she attended some groups, though she sat on the periphery. Prior to admission, fluoxetine 20 mg daily had been started and was continued during her stay. After four days on the unit, she reported an improvement in symptoms, including suicidal thoughts, and was discharged to outpatient care on a regimen of fluoxetine and hormone therapy.

## Discussion

Recent studies suggest that the prevalence of gender dysphoria is rising in children, adolescents, and adults, with rates estimated between 0.5% and 1.3% [3]. Similarly, selective mutism is relatively rare, with a point prevalence of around 0.03% to 1.9% [2]. Notably, selective mutism typically presents in younger children, before age 5, rather than in adolescents or young adults [2]. Social anxiety disorder is the most common comorbid condition with selective mutism, indicating a likely shared genetic risk [2]. A 2017 systematic review found that the most common anxiety disorders in transgender women were specific phobia (17%) and social anxiety disorder (9.8%), but it did not include any data on mutism [4]. The few case reports describing mutism in transgender patients have been limited to those with altered mental status, psychosis, or trauma, none of which apply to this patient [5-7].

In this case study, the patient developed mutism only after identifying as female in her twenties and reported feeling disgusted with the sound of her deeper-pitched voice. Although she had been previously diagnosed with social anxiety disorder, which is closely linked with selective mutism, her development of mutism later in life seems to be primarily driven by gender dysphoria. Her reluctance to speak appears to be tied to her discomfort with the mismatch between her expressed gender and the voice characteristics of her sex assigned at birth. While anxiety about how her voice sounds in social settings may have contributed to her development of mutism, it does not appear to be the primary factor. This patient’s mutism seems rooted in her own negative self-perception of her voice rather than a fear of social judgment. This distinction is important, as it suggests that her mutism is a symptom of gender dysphoria rather than a manifestation of selective mutism. In cases of selective mutism, an individual would not speak in social situations despite speaking in other contexts [2]. The differential diagnosis for selective mutism includes communication disorders such as speech sound disorder, as well as neurodevelopmental and psychotic disorders such as autism spectrum disorder or schizophrenia, none of which fit this patient’s presentation [2].

It is crucial to differentiate between these conditions, as mutism secondary to gender dysphoria requires different treatment than selective mutism. Selective mutism is typically treated with behavioral therapy, which gradually exposes an individual to speaking in increasingly difficult and anxiety-provoking situations, along with medications such as SSRIs [8]. In contrast, mutism related to gender dysphoria may be better managed with the addition of gender-affirming treatments such as vocal coaching or hormone therapy.

## Conclusions

In transgender individuals, voice can be a significant part of gender expression and identity, and the perception that one’s voice does not align with their gender identity may lead to mutism. While other disorders, such as social anxiety disorder and selective mutism, may be present in transgender individuals, it is important for clinicians to assess the etiology of mutism for possible signs of gender dysphoria in transgender patients. A thorough evaluation is essential to avoid misdiagnosis and ensure the patient receives appropriate treatment. More research is needed to clarify the incidence, progression, and treatment strategies for mutism in transgender patients. Gender-affirming treatments such as speech therapy and hormone therapy may further improve communication in transgender individuals with mutism.
